# Drain tube migration into the anastomotic site of an esophagojejunostomy for gastric small cell carcinoma: short report

**DOI:** 10.1186/1471-230X-10-50

**Published:** 2010-05-21

**Authors:** Peng-Sheng Lai, Chiao Lo, Long-Wei Lin, Po-Chu Lee

**Affiliations:** 1 Department of Surgery, National Taiwan University Hospital, Yunlin branch, Taiwan; 2 Department of Pathology, National Taiwan University Hospital, Yunlin branch, Taiwan

## Abstract

**Background:**

Intraluminal migration of a drain through an anastomotic site is a rare complication of gastric surgery.

**Case Presentation:**

We herein report the intraluminal migration of a drain placed after a lower esophagectomy and total gastrectomy with Roux-en-Y anastomosis for gastric small cell carcinoma. Persistent drainage was noted 1 month after surgery, and radiographic studies were consistent with drain tube migration. Endoscopy revealed the drain had migrated into the esophagojejunostomy anastomotic site. The drain was removed from outside of abdominal wound while observing the anastomotic site endoscopically. The patient was treated with suction via a nasogastric tube drain for 5 days, and thereafter had an uneventful recovery.

**Conclusions:**

Though drain tube migration is a rare occurrence, it should be considered in patients with persistent drainage who have undergone gastric surgery.

## Background

Intraluminal migration of a drain through an anastomotic site is a rare complication of gastric surgery [1]. The condition frequently presents with persistent drainage, and diagnosis is made by radiographic studies. We herein report the intraluminal migration of a drain placed after a lower esophagectomy and total gastrectomy with Roux-en-Y anastomosis for gastric small cell carcinoma. The drain was removed via the abdominal wound while the anastomosis was visualized endoscopically.

## Case Presentation

A 67-year-old man presented with a 6-week history of hiccupping and foul odor. He also complained of difficulty swallowing, diffuse epigastric pain, and weight loss. Panendoscopy revealed a large ulcerated mass at least 8 cm in diameter at the lesser curvature side of the anterior wall of the gastric upper body, with invasion into the lower esophagus. Biopsy was consistent with small cell carcinoma. Chest, abdominal and pelvic computed tomography (CT) showed distal esophageal involvement and lymphadenopathy, but no evidence of liver or distal metastasis. A lower esophagectomy, total gastrectomy with Roux-en-Y anastomosis, and feeding jejunostomy were performed. A 13-mm vacuum drain tube was inserted intraoperatively adjacent to the anastomotic site.

**Figure 1:**
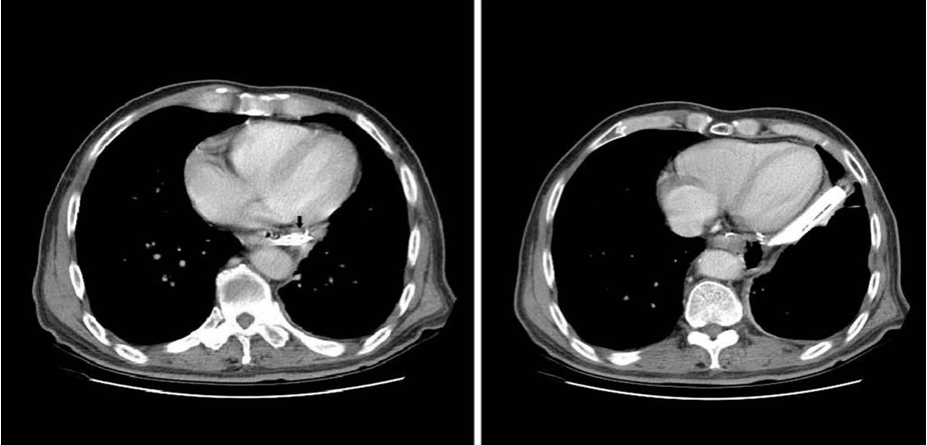
**Computed tomography indicates the possibility of drain tube migration into the esophagus (arrow)**.

**Figure 2:**
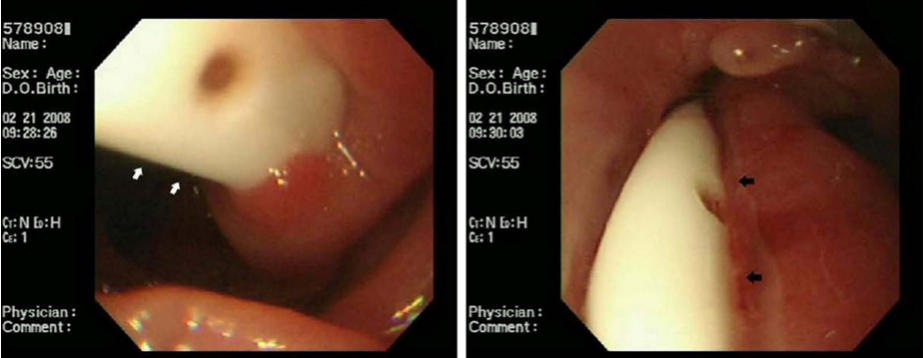
**Panendoscopy indicates that the drain had migrated into the esophagojejunostomy anastomotic site (arrow)**.

Postoperatively, approximately 80 ml of yellowish-white, foamy, foul smelling fluid was collecting from the drain tube each day. This drainage persisted for 1 month after surgery. CT was performed, and the result was consistent with drain tube migration (Figure 1). Panendoscopy indicated the drain had migrated into the esophagojejunostomy anastomotic site (Figure 2). The drain was removed from the outside of abdominal drain wound while observing the anastomotic site endoscopically. After the drain was removed, no intraperitoneal organs (such as small bowel, liver, or spleen) were visible. The mucosal defect over the anastomotic site caused by the migrated drain appeared to be well approximated after removal of the drain. The patient was treated with suction via a nasogastric tube drain for 5 days. He thereafter had an uneventful recovery.

The patient subsequently was treated with 4 cycles of combination chemotherapy consisting of cisplatin and etoposide (VP-16). Sixteen months after surgery the patient was alive and showed no signs of cancer recurrence. **The patient discussed in this report provided informed consent for all treatments rendered and consent for the publication of this case report and the use of radiological images**.

## Discussion

Anastomotic leakage after gastric surgery is a relatively common complication occurring in 10% to 15% of patients and carries a mortality of approximately 5% [2,3]. Management of leakage may be operative or non-operative, and most surgeons routinely place prophylactic drains at the anastomotic site during the initial surgery [4]. Persistent drainage is frequently the first sign of anastomotic leakage or drain migration [1]. The migration of a drain tube into the anastomotic site of an esophagojejunostomy is a rare complication after a gastrectomy [1].

Diagnosis of anastomotic leakage or drain migration is made based on contrast radiographic studies, i.e., evidence of leakage after radio-contrast examination may indicate anastomotic leakage or drain migration, as was present in our case [1]. It is uncertain how intraluminal migration of a drain may occur, though authors believe it may be a result of migration through the site of an anastomotic leak. In most cases, the drain is repositioned, either surgically or under radiographic guidance, and left in place as the anastomosis heals.

In the case presented herein, we left the drain in place because the color, amount, and odor of the drainage made us suspect there was minor leakage of the esophagojejunostomy anastomotic site. Because the amount of drainage did not decrease over the course of a month, we were concerned about the integrity of the anastomosis, and thus decided to check the esophagojejunostomy anastomotic site endoscopically. In this case, visualization of the anastomotic site endoscopically allowed us to confirm the integrity of the anastomosis after the drain was removed and determine that surgical correction was not required.

## Conclusion

In conclusion, intraluminal drain migration is a rare complication after gastric surgery, however, should be considered in cases where there is persistent drainage. Diagnosis is made by radiographic studies, and endoscopy offers an additional diagnostic modality.

## Consent

Written consent for publication was obtained from the patient.

## Competing interests

The authors declare that they have no competing interests.

## Authors' contributions

PSL and PCL participated in the surgery. CL helped to draft the manuscript. LWL performed the pathologic examination. All authors read and approved the final manuscript.

## Pre-publication history

The pre-publication history for this paper can be accessed here:

http://www.biomedcentral.com/1471-230X/10/50/prepub
